# Community Leaders’ Perspective of Strategies to Enhance Social Connectedness in Rural Communities

**DOI:** 10.1093/geroni/igab046.1230

**Published:** 2021-12-17

**Authors:** Patricia Oh, Len Kaye, Lori Parham

**Affiliations:** 1 UMaine Center on Aging, Bangor, Maine, United States; 2 University of Maine, Orono, Maine, United States; 3 AARP Maine, Portland, Maine, United States

## Abstract

Age-Friendly communities are charged with fostering a social environment where social connections are available, accessible, and meaningful. Thematic content analysis of 67 interviews (representing 73 communities) conducted between 12/09/2019 and 01/24/2020) and 59 interviews (representing 71 communities) conducted between 12/14/2020 and 1/19/2021 with age-friendly leaders in rural Maine suggested the importance of expanding multi-sectoral collaborations and developing flexible strategies that allow older people to create and maintain social connections, even during COVID. Prior to the pandemic, common strategies were: (1) local partners and volunteers; (2) in-person activities; (3) accessibility; (4) reciprocity; and, (5) neighborhood-specific solutions. During the pandemic, there was an increased reliance on regional partners and funders to develop low or no-tech and tech-enabled social opportunities. Additional adaptive strategies included: (1) intergenerational ties; (2) emphasizing fun; and, (3) flexibility. The study has implications for understanding how rural age-friendly communities develop and grow multi-sectoral collaborations to enhance social connections.

